# Postgraduate and research programmes in Medicine and Public Health in Rwanda: an exciting experience about training of human resources for health in a limited resources country

**DOI:** 10.11604/pamj.2016.23.171.8788

**Published:** 2016-04-08

**Authors:** Jean Baptiste Kakoma

**Affiliations:** 1 Faculty of Medicine and School of Public Health, University of Lubumbashi, Lubumbashi, DR Congo; 2 School of Medicine and School of Public Health / College of Medicine and Health Sciences, University of Rwanda, Kigali, Rwanda

**Keywords:** Postgraduate programme, research, medicine, public health, Rwanda

## Abstract

The area of Human Resources for Health (HRH) is the most critical challenge for the achievement of health related development goals in countries with limited resources. This is even exacerbated in a post conflict environment like Rwanda. The aim of this commentary is to report and share the genesis and outcomes of an exciting experience about training of qualified health workers in medicine and public health as well as setting - up of a research culture for the last nine years (2006 - 2014) in Rwanda. Many initiatives have been taken and concerned among others training of qualified health workers in medicine and public health. From 2006 to 2014, achievements were as follows: launching and organization of 8 Master of Medicine programmes (anesthesiology, family and community medicine, internal medicine, obstetrics & gynecology, otorhinolaryngology, pediatrics, psychiatry and surgery) and 4 Master programmes in public health (MPH, MSc Epidemiology, MSc Field Epidemiology & Laboratory Management, and Master in Hospital and Healthcare Administration); training to completion of more than 120 specialists in medicine, and 200 MPH, MSc Epidemiology, and MSc Field Epidemiology holders; revival of the Rwanda Medical Journal; organization of graduate research training (MPhil and PhD); 3 Master programmes in the pipeline (Global Health, Health Financing, and Supply Chain Management); partnerships with research institutions of great renown, which contributed to the reinforcement of the institutional research capacity and visibility towards excellence in leadership, accountability, and self sustainability. Even though there is still more to be achieved, the Rwanda experience about postgraduate and research programmes is inspiring through close interactions between main stakeholders. This is a must and could allow Rwanda to become one of the rare examples to other more well-to-do Sub - Saharan countries, should Rwanda carry on doing that.

## Commentary

The area of Human Resources for Health was undoubtedly one of the most critical challenges for the achievement of health and development goals. That is why the Global Health Workforce Alliance was created in 2006 to find a solution to the Human Resources for Health crisis [1]. This health workforce crisis is global and tremendously felt in post - conflict situations. Rwanda is one of the cases. To alleviate this challenge, many diversified initiatives have been taken and are still ongoing in the country. Among these are training of qualified health workers in medicine and public health as well as setting -up of a research culture. The aim of this commentary is to report and share the genesis and outcomes of an exciting experience about these for the last nine years (2006-2014) in Rwanda. The idea of organizing a postgraduate training in Rwanda was expressed in 1995 and 1996 in the framework of the Belgo -Rwandan cooperation in the health sector. The main reason was the shortage of qualified human resources for health after genocide. The training partly organized in Belgium was launched in May 1997. Two other intakes were recruited later on before the Rwandan government decided, in the course of 2004-2005, to run a new postgraduate programme completely organized in the country. There were two main reasons for this: high cost of training abroad and absolute certainty of directly benefiting from this training [2]. National University of Rwanda (NUR) faculty of medicine developed a multipronged 4-year postgraduate training project in the 5 priority specialties (anesthesiology, internal medicine, obstetrics and gynecology, pediatrics, and surgery). The programme was a synthesis of Eastern and Southern African masters programmes (Kenya, Tanzania, Zambia, Zimbabwe, and Uganda) with a more elaborated theoretical part to compensate gaps observed in the medical undergraduate programme [2].

Despite some human susceptibility and doubts about this new adventure, the process was launched in January 2006 due to the political will as regards the Rwandan Government's Vision 2020. Apart from the five permanent professors, several short-term teaching missions were spread out throughout the first three years: 50 visiting teachers came from different parts of the world (Belgium, Benin, Canada, Denmark, Kenya, Middle East, Senegal, South Africa, Sweden, Uganda, United Kingdom, and United States) to participate in this postgraduate training. Training mostly focused on class room teachings (75%) with less practical demonstrations and bedside teachings (25%). This was due to time constraints because almost all visitors were volunteers and full-time faculty in their own countries. Residents posted in different teaching hospitals (University Teaching Hospital -Butare, University Teaching Hospital -Kigali, and King Faisal Hospital, Kigali) were mentored by supervisors and consultants in the abovementioned hospitals that had been selected in order to allow annual rotations with three different scopes of training: rural, urban, and well equipped environments. Shortage of specialists in Rwanda was then very tremendous: in 2005, there were no more than 10 obstetricians, 10 pediatricians, 8 internists, 7 surgeons, and 4 anesthetists for almost 10 million people in Rwanda. Family and community medicine was subsequently included in the programme through a specific project in partnership with Colorado University. Other programmes followed (e.g. otorhinolaryngology, psychiatry). Outcomes for the new masters programme in medicine consisted also in allowing Rwandan postgraduates from the previous programme and those training abroad (European Union particularly, due to academic regulations) to take the final exam for graduation. At the end of Year 2010, a total of 18 candidates from previous intakes and 28 from the 2005-2006 intake were already graduated (8 in anesthesiology, 2 in dermatology, 12 in obstetrics and gynecology, 9 in internal medicine, 11 in pediatrics, and 4 in general surgery). Up to 2012, the total number of graduates was 90 specialists; and for the time being, the number has exceeded one hundred twenty.

National University of Rwanda School of Public Health was created in 2001 in order to provide leadership to address the health challenges the country was faced with. The Master in Public Health (MPH) programme was the first one to be organized under Tulane University's umbrella. Candidates were recruited among district hospital and health administration directors. Some years later, evening teachings were introduced, and the programme was open to a broad range of applicants. The Master of Science in Epidemiology (MSc Epidemiology) was introduced in 2005 through the French bilateral cooperation in partnership with “Ecole Nationale de Santé Publique” (i.e. National School of Public Health) of Nantes. Both programmes were run in a modular system for two years (8 modules) according to a part - time scheme. This associated with lack of prerequisites and close supervision proved to be a very tedious process so that graduation for successive intakes did not take place at the end of the second year. In 2008, the number of graduands and dysfunction reached the point where the school management structure was readjusted in an environment of severe staff shortage. The updated organizational and operating structure based on some core values (team work, ownership, performance based targets, and accountability) proved to be more efficient. In 2010, the school management urged all students with pending research projects to move forward with existing or allocated supervisors, and a regular monitoring and evaluation system was put into action. Results proved to be spectacular: 60 MPH and MSc Epidemiology students graduated at the special graduation ceremony organized on 15^th^ October 2010; this was more than the number that graduated during the 8 previous years. Clear and appropriate criteria were henceforth not only set up but also rigorously put into practice starting from academic year 2011. Therefore, the recruitment process included three preliminary steps before a deep and well conducted interview as the purpose was to get quality instead of gregarious attraction. On the whole, more than 200 holders of a master's degree graduated from the School of Public Health by 2014. In front of emergent and epizootic diseases, a Master's programme in Field Epidemiology and Laboratory management Training was launched in May 2010 in collaboration with CDC - Atlanta, African Field Epidemiology Network, and the Rwanda Center for Treatment and Research on AIDS, Malaria, Tuberculosis and other Epidemics. Up to now the programme has been a success: students are advantageously involved in all outbreaks occurring in the country since then, and 14 candidates of the first intake were graduated, followed by those of the second cohort.

Improvement of management and health care quality is one of the worrying issues in Rwanda besides the health staff shortage. That is why the Rwanda ministry of health decided to address along all these issues through an ambitious Human Resources for Health project. As a result, the school of public health in partnership with Yale University initiated in 2012 a Masters’ programme in Hospital and Healthcare Administration (MHA). This happened before the ongoing higher education reorganization as per the 2013 reform [3]. The school is now part of the College of Medicine and Health Sciences within the University of Rwanda and its attention is already attracted to next challenges. A curriculum for a Master's programme in Global Health Delivery has already been submitted to and approved by the Rwanda Higher Education Council; another one concerning Health Financing and Economics is in the pipeline. Meanwhile, a Master's programme in Supply Chain Management has been worked out through the East African Community Regional Center of Excellence on Supply Chain Management to be hosted by the school. In the early post-genocidal period, the priority objective of Rwanda was to train staff needed in various sectors of national life. Therefore, National University of Rwanda was asked to contribute to the development of the country in a more determining way and an environment characterized by a poor rate of lecturers holding a doctorate or equivalent degree (PhD, MMed…). A research commission was then created in 1998 and assigned several missions including among others that of defining a scientific research policy and strategies to produce and increase knowledge in order to address the multiple challenges that the country was facing, meanwhile developing postgraduate training in different fields [4]. Postgraduate training started in medicine, as abovementioned, and research was then an appreciable component of the curriculum. The two first PhD thesis presentations at National University of Rwanda were organized by National University of Rwanda faculty of medicine through a sandwich system with two Belgian universities (Gent University for a PhD in Medical Sciences / Physiology in January 2007; and “Université Libre de Bruxelles” for a PhD in Bio-Medical Sciences / Anatomy -Embryology in May 2007). In 2006, National University of Rwanda adopted a Scientific Research Policy [4]. From the beginning, its Research Commission was an active organ: very strong composition (skilled professors with strong background in research and research supervision), regular meetings, announcements and invitations to grants application, organization of annual research conferences, and publishing of “Etudes Rwandaises” (Rwandese Studies). As for peripheral activities, the organizational structure was only operational at the faculty of medicine. Meanwhile the central managerial structure became more and more organized so that there were since 2007: a Research, Consultancy and Technology Transfer Committee (RCTT-C), a Standing Committee for Graduate Research Degrees (SC-GRD), and a Research Screening and Ethics Clearance Committee (RSEC-C). The challenge was still huge as the peripheral level seemed to drag its feet.

National University of Rwanda faculty of medicine initiated an encouraging research culture that became a mainstream attitude. Research activities were organized in bygone days through an incentive structure called “Cellule de Recherche” (Research-Cell) and later on the vice deanship in charge of postgraduate studies and research, departmental research commissions and weekly meetings at faculty level to discuss research protocols (for MMed and PhD degrees, applications to grants…). These activities acumen culminated every year in a scientific meeting with national or international impact called “Journées Médicales de Butare” (Butare Medical Conference). In order to crystallize efforts, the meeting was always thematic and focused on restricted topics: Infectious Diseases (2005); HIV / AIDS, Tuberculosis, Malaria, and Medical Education (2006); Cancers in Rwanda (2007); and Diabetes in Rwanda (2008). The main objective was not only to maintain a research culture among people mostly dealing with their primary occupations (teaching and healthcare) but also to manage heaps of data available across the country in order to get diseases baselines through a more comprehensive knowledge. It is worth mentioning that the faculty of medicine was the first health institution in Rwanda to be interested in Non Communicable Diseases when HIV / AIDS, malaria, and tuberculosis were the only celebrated diseases in the country! The PhD sandwich programme in partnership with Swedish higher education and research institutions (Uppsala, Göteborg, and Karolinska Institutet) through National University of Rwanda -Swedish International Development Cooperation Agency Project contributed also to strengthen the research culture. It took a long time before National University of Rwanda School of Public Health realized that lack of evidence-based publications was the main reason of less scientific visibility of Rwanda despite the monumental mass of data collected from the ground. The school consequently decided to undergo a radical sloughing to polish up its image and become a credible partner of world class institutions. Three factors contributed to this: a shown and well deserved self-confidence, a renovated organizational structure, key and polished partnerships. It is well known that “at the beginning, is willpower!” and to be efficient, this willpower should stand on well directed and recognized skills. So, the School of Public Health decided to make use of any means and allocated specific tasks to each member in accordance to his / her scientific qualifications (e.g. research protocols - writing and research supervisions, application to grants, active participation in research teams and workshops, writing up abstracts and making posters, active participation in national and international conferences, publishing and editing special medical journal issues -i.e. Rwanda Medical Journal, presentation of concept notes for PhD application, PhD supervision). The school also initiated since 2010 advanced postgraduate research trainings in public health including a Master of Philosophy (MPhil) and PhD programmes. Besides, a new era of well-diversified partnership with renowned organizations (e.g. CDC, Rockefeller Foundation, Duke Doris Charitable Foundation, Belgian Technical Cooperation, and Training Health Researchers into Vocational Excellence in East Africa Consortium) indubitably contributed to the reinforcement of the institutional research capacity and visibility. This allowed the school to excel in leadership and accountability towards self sustainability.

In-service training for health professionals (Continuing Professional Development / CPD being one of the aspects) has become a wide and familiar exercise in Rwanda. In spite of this and as far as human resources for health are concerned, the challenge is still tremendous. After the 12 Kampala Calls to Action (2008), 39 African countries (and most of them sub-Saharan) were still part of the 57 countries with a critical health workforce shortage or Human Resources for Health crisis countries [5]. The trend is now towards an increase due to Rwanda Government support in order to strengthen undergraduate and postgraduate programmes in health sciences into the country. Besides, research is officially encouraged through the ministry of health. This brings in two advantages: acquirement of more scientific visibility, and promotion of evidence-based practice beneficial to the country and other regions. In conclusion, it is a pleasure as well as a life satisfaction to participate in a building project of human resources for health like it was and is still happening in Rwanda, and be involved into exciting interactions between main stakeholders, i.e. ministry of health, partners in the health sector, and concerned higher education institutions. In this area, Rwanda could be an example to other more well-to-do sub Saharan countries, should Rwanda carry on doing that.
